# Clinical Study to Individual Treatment for Major Aortopulmonary Collaterals of Tetralogy of Fallot

**DOI:** 10.1155/2019/1603712

**Published:** 2019-05-15

**Authors:** Qing Guan, Jiarong Li, Kai Deng, Xiaoming Wu, Shiyuan Tang, Chengming Fan, Xun Wu, Shuwen Yuan, Jinfu Yang

**Affiliations:** ^1^Department of the Cardiovascular Surgery, The Second Xiangya Hospital, Central South University, Middle Renmin Road 139, 410011 Changsha, China; ^2^Department of Radiology, The Second Xiangya Hospital, Central South University, Middle Renmin Road 139, 410011 Changsha, China

## Abstract

**Objectives:**

To build a guideline for the individual treatment of Tetralogy of Fallot (TOF) with major aortopulmonary collaterals (MAPCAs) and tentatively establish the occlusion index of MAPCAs.

**Methods:**

According to the diameter of the aortopulmonary collaterals (R: mm) and the bodyweight of the children (weight: kg), K= ((∑*R*^2^)/*Wt*) was set as the occlusion index of TOF with MAPCAs. A retrospective study was initially performed in 171 patients who suffered from TOF with MAPCAs and underwent cardiac malformation repair to investigate the intervals of the K value: K≥2, 1<K<2, and K≤1. In order to examine the reliability of the intervals derived from the retrospective study, a prospective study was conducted in the following 209 cases. When K≥2, the collaterals occlusion was performed immediately behind surgical corrections. The postoperative condition changes in patients with 1<K<2 were observed first and managed by extending mechanical ventilation, while taking further treatments as their conditions worsen. As for patients with K≤1, no occlusion was performed. Finally, the circumstances of collaterals occlusion, postoperative ventilator assist time, and ICU resident time were collected and analyzed.

**Result:**

The proportion of the patients treated with occlusion and the postoperative ICU resident time (p<0.05) in patients with 1<K<2 in the prospective study did dramatically decrease when compared with those of the retrospective studies.

**Conclusion:**

Due to restrictions on medical conditions in China with a large population base, a standard individual treatment of TOF with MAPCAs should be established based on the Aortopulmonary Collaterals Occlusion Index K= ((∑*R*^2^)/*Wt*), which can effectively avoid unnecessary collateral occlusion, minimize trauma, and shorten the length of ICU and hospital stay. When K≥2, the collateral occlusion and surgical correction are recommended to be performed simultaneously. When 1<K<2, whether to occlude collaterals depends on the patients' postoperative conditions with extending ventilator time. When K≤1, do not deal with collaterals.

## 1. Introduction

Tetralogy of Fallot (TOF) with major aortopulmonary collaterals (MAPCAs) is a well-known and severe congenital heart disease which always confused medical staff. During radical correction of TOF, if MAPCSs are not treated, severe pulmonary circulation congestion and systemic circulation steal will occur postoperatively [1]. It always requires a longer period of mechanical ventilation to maintain the stability of vital signs such as breathing and circulation. Many patients have complications such as low cardiac output syndrome, bloody sputum, lung infection, and pleural effusion [2]. Transcatheter occlusion of MAPCA after surgical correction of TOF effectively solves this problem [3]. However, most previous doctors completely principally relied on experience in the treatment of MAPCAs. There is no specific standard for transcatheter collaterals occlusion. Based on this, according to the treatment of TOF patients with aortopulmonary collaterals in our hospital for the past 10 years, we have initially proposed the collaterals occlusion index to provide a theoretical basis for the individual treatment of the disease.

## 2. Data and Methods

### 2.1. Clinical Data

We counted 380 cases of patients who were identified TOF with MAPCAs by echocardiography and CT angiography (CTA) preoperatively and underwent radical correction of TOF from the January 2008 to March 2018 at The Second Xiangya Hospital. These patients account for 28.1% of our total TOF operations (1351 cases) during the corresponding period, and the remaining 71.9% of patients (971 cases) without obvious aortopulmonary collaterals did not belong to this research. Patients with a double outlet of the right ventricle, TOF with pulmonary atresia, or TOF with prior palliative shunts were excluded. The cases of routine ligation of PDA during surgery did not belong to the category of collaterals described in this article. Among these, there are 171 cases in the retrospective study, there are 209 cases in the prospective study, and there are 213 males and 167 females. Ages ranged from 6 months to 58 months. The mean age was 22 months. Weights ranged from 5.0 kg to 14.8 kg with an average of 9.8 kg. To demonstrate that all patients in our study reached the standard of radical correction, we also counted McGoon values of them which ranged from 1.1 to 2.15 and averaged 1.52. The collateral diameters (R: mm) were measured by cardiac CTA, and the accurate statistics of aortopulmonary collaterals with a diameter of 1 mm or more were obtained. The number of collaterals per patient ranged from 1 to 6 and the collaterals diameter ranged from 1 to 5.2 mm. The average number of collaterals of K≥2 was 4.1, the average number of collaterals of 1<K<2 was 3.3, and the average number of collaterals of K<1 was 2.5.

### 2.2. Method

Based on the diameter (R) of the collaterals and the weight (kg) of the children, the occlusion index K= ((∑*R*^2^)/*Wt*) mm^2^/kg was set. In the retrospective study, we summarized the clinical information of 171 patients who attended our hospital before the year 2012 and divided the cases into three intervals: K≥2, 1<K<2, and K≤1. After 2012, in order to examine the reliability of the intervals derived from the retrospective study, we conducted a prospective study of 209 patients according to the occlusion index. Patients with K≥2 were all treated with transcatheter occlusion immediately at the end of surgical correction of TOF. Patients with 1<K<2 firstly accepted prolonged mechanical ventilation time after surgery. Only if bloody sputum, low cardiac output, hypoxemia, oliguria and hyperlactatemia, or other complications occurred, collateral occlusion would be applied in the following. While there was a change in the condition of patients with K≤1, it was a priority to consider other possible factors and the related treatment was performed, and none of them received occlusion operation after radical correction of TOF. We counted the number and proportion of patients with transcatheter collateral occlusion in each interval and collected ventilator assist time and ICU resident time to contrast the prospective study with the retrospective study.

**Figure 1:**
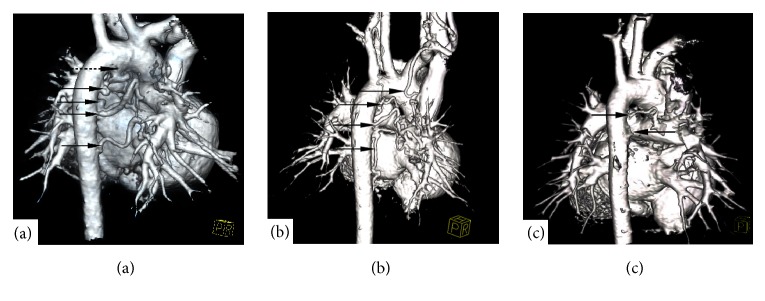
CTA images of 3 typical patients with MAPCAs of different K values (collaterals marked by arrows): (a) the patient with K≥2 (2.28), and one large collateral was ligated during operation (dotted arrow). (b) The patient with 1<K<2 (1.60). (c) The patient with K≤1 (0.62).

**Figure 2:**
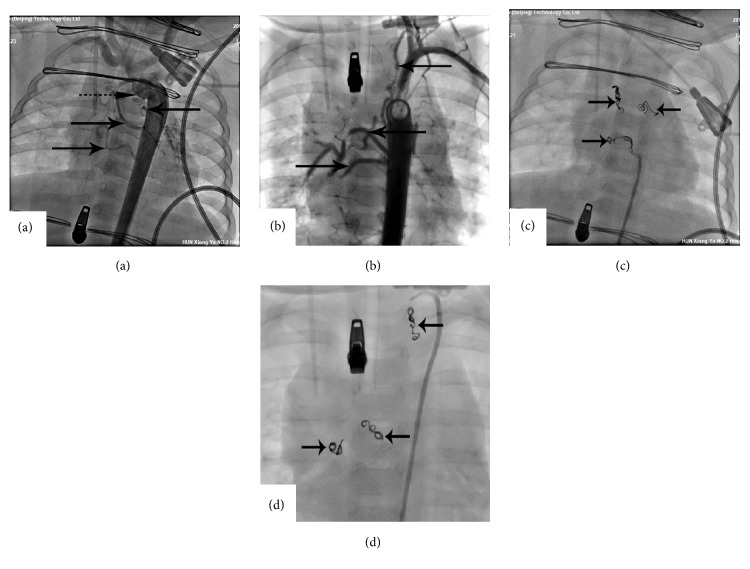
The angiograms of the typical patients before and after transcatheter occlusion. (a) The patient with K≥2 before the occlusion, and one collateral had been ligated before (dotted arrow). (b) The patient with 1<K<2 before the occlusion. (c, d) completed occlusion of the MAPCAs.

Figure 1 exhibits preoperative typical cardiac CTA images of three patients with MAPCAs in different collateral occlusion index intervals and Figure 2 is two of the three patients angiocardiography images before and after occlusion.  Figure 1(a) is a cardiac CTA of an 8-month-old and 7 kg boy with K≥2 (2.28) whose five large MAPCAs originated from the aorta supplying blood to the left and right pulmonary artery (arrows in Figure 1(a)) and including one collateral arisen from aortic arch which was ligated during surgery operation (dotted arrow). Postoperative angiocardiography verified the diagnosis and three large collaterals were occluded immediately (Figures 2(a) and 2(c)).  Figure 1(b) shows the CTA image of a 2-year-old and 10 kg boy with 1<K<2 (1.60) whose beginning of the thoracic aorta, abdominal aorta, and right subclavian artery gave out large aortopulmonary collaterals supplying blood to pulmonary arteries (indicated by arrows). Collateral occlusion was performed four days after the operation of TOF (Figures 2(b) and 2(d)).  Figure 1(c) is a cardiac CTA image of a 13-year-old and 13kg girl with K<1. There were two collaterals originated from descending aorta (arrows in Figure 1(c)) involved in abnormal blood supply and the transcatheter angiography was not required for the occlusion index was just 0.62.

### 2.3. Data Analysis

Descriptive analyses were conducted by using IBM SPSS Statistics software for Mac version 24.0 (SPSS Inc., Chicago, IL, USA). Continuous variable (ventilator assist time and ICU resident time) was presented as the mean ± standard deviation (SD). Normality test, homogeneity test, Student's t-test, and approximate t-test were conducted to identify the difference between retrospective study and prospective study. P<0.05 was considered statistically significant.

## 3. Result

### 3.1. Overview

A total of 380 cases of TOF radical correction combined with aortopulmonary collaterals were included, and 78 cases were performed transcatheter collaterals occlusion in total (Table 1). In the retrospective study, 39 cases were performed occlusion. All the 19 patients with K≥2 were performed collateral occlusion. A case had severe postoperative complications like hyperlactatemia, respiratory acidosis, severe lung infection, and difficult ventilator weaning only if tracheotomy. Another one with unbalanced pulmonary arteries development and larger aortopulmonary collaterals died of low cardiac output syndrome on the 4th day after occlusion. 23 of 47 patients with 1<K<2 had varying degrees of postoperative complications such as heart failure, bloody sputum, hypoxemia, and hyperlactatemia, where 5 cases recovered by prolonging mechanical ventilation for more than 40 hours and 18 cases got collateral occlusion finally. In the 105 cases with K≤1, 2 cases were performed collateral occlusion due to low cardiac output and hypoxemia after surgery, but the improvement of symptoms was not obvious, because it is mainly due to the small size left ventricle (1 case) or tracheal bronchus (1 case). With adjusting the vasoactive drugs, parameters of mechanical ventilation, and prolonging mechanical ventilation time, the patients gradually recovered. The rest of the patients recovered successfully. At prospective study of 209 cases, all the 24 cases with K≥2 and 15 of 63 cases with 1<K<2 were performed occlusion; however, all 122 cases with K≤1 without occlusion. Only one patient with K≥2, treated with occlusion after operation immediately, suffered low cardiac output syndrome for a small left ventricle, but we weaned him off the ventilator after 82 hours postoperatively via adjusting vasoactive drugs and extending ventilator-assisted time. Among the 63 patients with 1<K<2, 32 patients had various severe complications such as low cardiac output, bloody sputum, hyperlactatemia, and oliguria. Those complications in 17 cases were resolved just via long ventilator time and some other symptomatic treatments. More severe symptoms occurred in the other 15 cases with extending mechanical ventilation. As transcatheter occlusions had to be performed, all of them got well. 1 patient in K≤1 who was due to diaphragmatic muscle dysfunction had difficulty in ventilator weaning after the correction. Via receiving a tracheotomy, sitting position, intermittent weaning, and other treatments, the child was finally weaned off ventilator 45 days postoperatively.

### 3.2. Decrease in Occlusion Rate

Compared with group of patients with 1<K<2 at the retrospective study, where 18 of 47 (38.3%) cases were treated with collateral occlusion and the 18 patients' average K value was 1.63, we found that the occlusion ratio at prospective study was significantly decreased under the guidance of the collateral occlusion index K((∑*R*^2^)/*Wt*). Only 15 of 63 cases had collateral occlusion, in which the occlusion rate was 23.8% and the 15 patients' average K value was 1.87.

### 3.3. Reduction in ICU Resident Time

After setting the transcatheter collateral occlusion index, according to the K value, we standardized the treatment of parents avoiding unnecessary angiocardiography and transcatheter collateral occlusion, also timely treated patients requiring occlusion. Compared with the retrospective study, because there were more patients with 1<K<2 in the prospective study treated with extending the mechanical ventilation time in the early postoperative period, this part of patients' average mechanical ventilator time increased slightly (P >0.05). But the average ICU resident time was significantly shortened (P <0.05) (Table 2).

## 4. Discussion

The aortopulmonary collateral is one of the common factors influencing the effect of radical correction for TOF [4]. In the early stage, cardiac surgeons did not know so much about collaterals and did not treat the collaterals when performing TOF radical correction. With the development of medical technology and computed tomography imaging technology, cardiac surgeon gradually has advanced understanding about the aortopulmonary collaterals, and the treatment of collaterals has become an important part of radical correction of TOF [5]. As aortopulmonary collaterals are variable and the surgical field is relatively fixed, though ligating collaterals during operation directly is the most economical method, the exposure and ligation of collateral vessels is a very tough operation which always creates big wounds, easily damages surrounding tissue, and consumes time. There is also the possibility of being unable to find all collaterals [6]. Transcatheter occlusion, which preferably solves the disadvantages of ligating, is becoming a popular method for treating collaterals in recent years. The occlusion can be performed before, during, or after operation [7]. In the past, the method was always delivered to the cardiac catheterization room for collaterals occlusion, while the large medical institution has a one-stop hybrid operating room, which can occlude collaterals after surgical operation at once [8]. Most research on the management of MAPCAs has focused on occlusion of MAPCAs before surgical correction of TOF; however, occlusion of MAPCAs before surgical correction could lead to a further decrease in arterial oxygen saturation, and the patient needed surgical correction immediately after transcatheter closure of MAPCAs [9]. Moreover, most cardiac centers in China are not equipped with a complete hybrid operating room for the restriction of medical conditions, so most of the surgeons might prefer surgical correction firstly and then depend on the postoperative condition to determine whether to conduct occlusion [10]. But there were no related documents to suggest the indication of occlusion, and cardiac surgeons might blindly treat it based on their personal experience.

Our center is one of the biggest ones of China. During the past decade, there were more than 1300 surgeries for pediatric congenital heart disease and 130 surgeries for TOF were performed each year. According to incomplete statistics, the total number of patients meeting our research is 1351.

In our former study, we proposed to set collateral diameter-to-body weight ratio as the measure of whether further treatment of aortopulmonary collaterals is needed and provided preliminary ideas for the treatment of MAPCAs [11]. In the further study, we found that the collaterals are always multiple and different in diameter. The collaterals should or not be treated after the correction is more closely related to the cross-sectional area of total collaterals. When the sum of diameters and the number of collaterals are fixed, the smaller the difference between each collateral diameter is, the smaller the total cross-sectional area will be, and the split-flow of blood will also be fewer. Vice versa, single collateral may have a greater blood flow. Therefore, we further analyzed the results of the first phase (before 2012) and proposed to set up a better “Occlusion Index” based on the relationship between the cross-sectional area of the collaterals and body weight; among the 171 patients in the retrospective study, 19 patients with K≥2 were all treated with collateral occlusion and 18 of 47 cases with 1<K<2 were treated with occlusion. Although 2 of the 105 cases with K≤1 received occlusion, it did not significantly shorten the natural process of disease, for the exact cause was the small left ventricular or right upper lobe tracheal bronchus. After the year 2012, we conducted a prospective study with 209 cases based on the division of K value we had defined and divided the patients into three groups: K≥2 group, 1<K<2 group, and K≤1 group. As what we had planned, transcatheter occlusion was immediately conducted to patients with K≥2 after correction of TOF and none of the 122 cases with K≤1 was treated with further treatment. 15 of 63 cases with 1<K<2 were managed by occlusion because of occurrence of severe bloody sputum and/or low cardiac output syndrome. Hence, the biggest change of the occlusion situations above the three groups was the patients with 1<K<2 and this group was exactly the one which always puzzled surgeons. With the setting of standard treatment which based on the Occlusion Index, more patients with 1<K<2 just needed to adjust vasoactive drugs or prolong ventilator time to improve the symptoms, instead of collateral occlusion. Compared with the retrospective study, the increase of average ventilator time in the prospective study was certainly slight and negligible (P >0.05), while the average ICU resident time did decrease significantly (P <0.05). Because, in the prospective study, more patients were free from the risk of trauma and infection caused by transcatheter occlusion. Less occlusion also helped to reduce the time of anesthesia and reduced the inhibition of breathing, circulation, digestion, and other systems caused by anesthesia. So it is accessible that the average ICU resident time cut down obviously, to some extent, saving medical resources and reducing costs.

Analyzing the differences between retrospective and prospective study, we found that in the patients of 1<K<2 in retrospective study the occlusion rate was higher and the average K value of patients with occlusion was lower (1.63), while the average K value of the patients treated with occlusion in prospective studies was 1.87. The result indicates that more patients underwent collateral occlusion in the same disease condition in the past. It can be speculated that most of the cardiac surgeons performed the aortopulmonary collaterals aggressively. When the condition worsens, the first choice may be occlusion instead of sufficient mechanical ventilation time, which can be seen in not only the ones with 1<K<2, but also the ones with K<1, showing active excessively and blind, and sometimes delaying the treatment of the real cause. Such as the two cases with K<1 mentioned in the retrospective study, most of these patients' complications were always due to small left ventricular, high pulmonary vascular resistance, or an incomplete correction, instead of collaterals. Even if occlusion was performed, the natural course of disease remained roughly. There were also a few of doctors who are relatively conservative. Postoperatively standing period of ventilator-assistance might cause some of the collaterals supposed to be intervened still pathogenic, delaying the best time for occlusion and causing a poor prognosis. Therefore, it is very important to establish an index for the occlusion of MAPCAs and standardize the treatment indications of the collaterals after surgical correction. In the actual observation in the interval of 1<K<2, we also found that, as the K value was the same, with the increase of body weight of children, the possibility of requiring occlusion would increase. And in the interval of 1<K<2, further treatment for the patients with single collateral was more necessary than for the ones with multiple collaterals.

## 5. Conclusion

In summary, we believe that the collateral occlusion index K=((∑*R*^2^)/*Wt*) has preliminary guiding value for the major aortopulmonary collateral management after correction of TOF, which can effectively avoid unnecessary collateral occlusion, minimize trauma, and shorten the length of ICU and hospital stay. According to the collateral occlusion index surgeons can treat the aortopulmonary collaterals according to the concrete situation at the right moment. When K≥2, the collaterals are supposed to be treated simultaneously with radical surgery. When 1<K<2, the first step is close observation with extending ventilator time and the collaterals are treated depending on the decline of illness condition. When K≤1, the collaterals should not be treated, and considering other reasons firstly if there is a change in the condition to avoid unnecessary treatment and delay of the treatment of the real cause is desirable.

## Figures and Tables

**Table 1:** Collateral occlusion situation between retrospective study and prospective study based on the different parts of K value.

	Total (n)	Retrospective study	Ratio (%)	Total (n)	Prospective study	Ratio (%)
		Transcatheter occlusion cases (n)			Transcatheter occlusion cases (n)	
K2	19	19	100%	24	24	100%
1<K<2	47	18	38.3%	63	15	23.8%
K1	105	2	1.9%	122	0	0

**Table 2:** Comparison of ventilator assist time and ICU resident time of parents with 1<K<2 in retrospective study and prospective study.

	ventilator assist time (h)	ICU resident time (d)
Retrospective study (n=47)	48.5 ±12.3	5.3±2.2
Prospective study (n=63)	49.5±7.5	4.1±1.6
P value	0.62	0.003

## Data Availability

The images and statistics data used to support the findings of this study are available from the corresponding author upon request. Part of those is from previously reported studies and datasets, which have been cited at relevant places within the text as Reference [11].
